# Thalidomide-Revisited: Are COVID-19 Patients Going to Be the Latest Victims of Yet Another Theoretical Drug-Repurposing?

**DOI:** 10.3389/fimmu.2020.01248

**Published:** 2020-05-28

**Authors:** Athar Khalil, Amina Kamar, Georges Nemer

**Affiliations:** ^1^Department or Biochemistry and Molecular Genetics, American University of Beirut, Beirut, Lebanon; ^2^Vascular Medicine Program, Department of Internal Medicine, American University of Beirut, Beirut, Lebanon; ^3^Genomics and Translational Biomedicine, College of Health and Life Sciences, Hamad Bin Khalifa University, Doha, Qatar

**Keywords:** COVID-19, cytokine storm, lung injury, thalidomide, anti-inflammatory drug

## Abstract

The coronavirus disease 2019 (COVID-19) pandemic is a worldwide threatening health issue. The progression of this viral infection occurs in the airways of the lungs with an exaggerated inflammatory response referred to as the “cytokine storm” that can lead to lethal lung injuries. In the absence of an effective anti-viral molecule and until the formulation of a successful vaccine, anti-inflammatory drugs might offer a complementary tool for controlling the associated complications of COVID-19 and thus decreasing the subsequent fatalities. Drug repurposing for several molecules has emerged as a rapid temporary solution for COVID-19. Among these drugs is Thalidomide; a historically emblematic controversial molecule that harbors an FDA approval for treating erythema nodosum leprosum (ENL) and multiple myeloma (MM). Based on just one-case report that presented positive outcomes in a patient treated amongst others with Thalidomide, two clinical trials on the efficacy and safety of Thalidomide in treating severe respiratory complications in COVID-19 patients were registered. Yet, the absence of substantial evidence on Thalidomide usage in that context along with the discontinued studies on the efficiency of this drug in similar pulmonary diseases, might cause a significant obstacle for carrying out further clinical evaluations. Herein, we will discuss the theoretical effectiveness of Thalidomide in attenuating inflammatory complications that are encountered in COVID-19 patients while pinpointing the lack of the needed evidences to move forward with this drug.

## Introduction

The sudden epidemic outbreak of the new coronavirus disease 2019 (COVID-19) in Wu Han City, China, has rapidly spread all over the world, leading to one of the worst pandemic outbreaks since the Spanish Flu that occurred 100 years ago (1). The culprit infectious pathogen, which causes severe acute respiratory syndrome (SARS), is yet another coronavirus (SARS-CoV-2) that is very similar to the previous viruses that caused the epidemic SARS in 2003 and MERS (Middle-Est Respiratory Syndrome) in 2012 (2). This highly contagious disease has spread throughout China and reached around 200 other countries within 2 months only (3). Based on that, the World Health Organization (WHO) declared the COVID-19 outbreak a pandemic on March 11th 2020. Till May 1st 2020, the confirmed number of cases surpassed 3.5 millions and resulted in more than 250,000 deaths across the globe (4). Fortunately, the severity of this disease is only encountered in about 20% of the cases where the patients develop respiratory failure, septic shock, and multi-organ dysfunction. According to the data reported so far, older adults, particularly those with severe underlying health conditions, are more prone to acute inflammatory reactions and lethal manifestations of this viral infection (3). Herein, we will discuss the pathological progression of this disease along with the activated inflammatory response that underlies the lethal complications of COVID-19. We will also evaluate the current status of Thalidomide usage as an anti-inflammatory therapy for COVID-19 induced pneumonia and acute lung injury (ALI).

## COVID-19 and the Cytokine Storm: a Role for Anti-Inflammatory Drugs in the Treatment?

Since the human respiratory system is the primary target for coronavirus pathogens, abnormal respiratory findings are highly detected in COVID-19 patients. The initial pulmonary symptoms include dry cough and coarse breathing sounds of both lungs (5). The progression of this infection starts with mild manifestations in the lungs, including (a) edema (b) proteinaceous exudate with globules (c) patchy inflammatory cellular infiltration, and (d) moderate formation of hyaline membranes (6). In more advanced cases, pulmonary ground-glass changes are accompanied by bilateral diffuse alveolar damage with edema, pneumocyte desquamation, hyaline membrane formation, interstitial lymphocyte infiltration, and multinucleated syncytial cells in the lungs (7, 8). At the site of injury, extensive infiltration of neutrophils, and macrophages is detected among patients with severe infection. Similarly, an increased number of neutrophils and monocytes is encountered in their peripheral blood while a suppressed cell count of CD4 and CD8 T and natural killer (NK) cells is reported (3, 9).

The uncontrolled release of pro-inflammatory cytokines named as the “cytokine storm,” starts initially in the immunopathological lungs and spreads throughout the body via the systemic circulation (10). This cytokine storm initiates lung injures and is considered the primary clinical cause of death among COVID-19 patients (11). With the accompanied exaggerated response from both T-cells and macrophages, this event can cause apoptosis of the epithelial and endothelial cells leading to lethal acute lung injury. Among the highly induced pro-inflammatory cytokines that are elevated in the epithelial cells of patients' airways and are involved in enhancing the oxidative stress status are: interleukin (IL)-1β, IL-2, IL-6, IL-8, tumor necrosis factor-alpha (TNF-α), and interferon alpha/beta (IFN-α/β) (7). Usually, this will be followed by an extrapulmonary systemic hyper inflammation syndrome that can lead to vascular hyperpermeability and eventually to multiple organ failure (12). Thus, if kept untreated, COVID-19 can cause damage to the heart, the liver, and the kidneys, as well as to organ systems such as the blood and the immune system (13). The resultant multi-organ damage is mainly caused by the upregulated circulating cytokines and the overexpression of inflammatory mediators in the interstitial space of various organs that induce universal endothelium and parenchyma injuries (14–16).

Although this viral infection might be hypothetically curbed only by anti-viral and respiratory supportive therapies yet, the cytokine storm presents a severe challenge to the body and should also be tackled using anti-inflammatory drugs (3). Since immunotherapeutic approaches can be involved in targeting inflammatory mediators and in neutralizing passively the SARS-CoV2 or preventing its entry to the host, evaluation of their usage as an adjunct therapy in severe cases is being considered (17) As such, drug repositioning for several known anti-inflammatory and immunomodulatory drugs has emerged as a rapid approach to reduce the fatalities in the last months. The advantages of drug repositioning strategies rely mainly on the low cost, the reduced time to reach the market, and the existence of pharmaceutical supply chains for formulating and distribution (18). Among the tested anti-inflammatory drugs are the non-steroidal anti-inflammatory (NSAID) drugs, glucocorticoids, intravenous immunoglobulin therapy, NK cell-based immunotherapy, immunosuppressants, and inflammatory cytokines antagonists (17, 19). Although some of these drugs have shown to be efficient in COVID-19 treatment, yet the accompanying adverse side effects or the reported non-significant outcomes did not support their further usage (4, 20, 21). So far, chloroquine and hydroxychloroquine usage have been highly applauded and was given an emergency approval by the FDA to slow the progression of COVID-19 among critical cases. Yet, the anti-viral and anti-inflammatory effects of these drugs require more clinical and pre-clinical studies to confirm their effectiveness and to rule out any associated severe side effects that might limit their usage (3).

## Thalidomide Between the Past and the Present

Sixty years ago, the medical usage of a novel synthetic glutamic-acid derivative termed Thalidomide [α-(N-phthalimido glutarimide)] resulted in a tragedy of birth defects that was never encountered before. This drug was developed in Germany and was distributed to 46 different countries as a sedative drug for treating morning sickness in pregnant women (22). From the time Thalidomide was marketed in 1957 till the date of its withdrawal in 1961, over 10,000 children were affected with severe congenital deformities including stunted limb development, cleft lip and palate, abnormal eyes and ears, and congenital heart diseases (23). Back then, the safety of Thalidomide was only confirmed in rodent models. Conversely, it was not approved by the FDA due to the reported associated peripheral neuropathy in adults (24). This drug pinpointed for the first time on the existence of species-specificity in reaction to medications and caused a remarkable shift in drug testing strategies.

Although Thalidomide was removed from the market in the 1961, research studies continued to test its effectiveness in other conditions, including autoimmune disorders, such as chronic graft vs. host disease and rheumatoid arthritis (25). Moreover, its efficacy was evaluated in several dermatologic conditions, including aphthous stomatitis, Behçet's syndrome, lupus erythematosus, prurigo nodularis, Kaposi's sarcoma, pyoderma gangrenosum, and lichen planus (26, 27). The promising reported results encouraged further testing of this drug in treating tuberculosis, human immunodeficiency viruses (HIV), and several cancers like multiple myeloma, glioblastoma, prostate, and lung cancer (26, 28). While the outcomes varied between the tested diseases, the only remarkable success was confirmed in treating Erythema nodosum leprosum (ENL) and multiple myeloma (MM) which guaranteed Thalidomide FDA approval as a treatment of choice for these two conditions in 1998 and 2006, respectively (Table 1) (29). However, due to its known serious teratogenicity, the prescription and utilization of this drug are still under strict control by the System for Thalidomide Education and Prescribing Safety (STEPS) program that monitors prescribing, dispensing, and usage of this drug (25). The reason behind this restricted precautious usage of Thalidomide is mainly linked to the yet unresolved mechanism(s) of action, whether in treating these diseases or in triggering congenital malformations (23). So far, among the most accepted mechanisms are those related to its effect on (1) DNA replication or transcription, (2) synthesis and/or function of growth factors, (3) inhibition of cell adhesion molecules, (4) modulation of the immune response, (5) chondrogenesis, nerve/neural crest toxicity, (6) suppression of angiogenesis, and (7) cell death or injury (30, 31).

**Table 1:** The latest level of studies on Thalidomide usage in several conditions/diseases.

**Diseases/conditions tested for Thalidomide usage**	**Level of studies**
Morning sickness	Discontinued due to its reported teratogenicity/1961
Multiple myeloma	FDA approval/2006
Erythema nodosum leprosum	FDA approval/1998
Crohn Disease	Clinical trial level/recruiting
Myelofibrosis	Clinical trial level/recruiting
Thalassemia	Clinical trial level/recruiting
Idiopathic pulmonary fibrosis	Clinical trial level/completed
Psoriasis, plaque-type	Clinical trial level/completed
HIV infections	Clinical trial level/completed
Graft vs. host disease	Clinical trial level/completed
H1N1-induced pneumonia	Pre-clinical level/mice model
Paraquat (PQ) induced pulmonary inflammation and fibrosis	Pre-clinical level/mice model
Acute lung inflammation by *Klebsiella pneumoniae*	Pre-clinical level/mice model

## The Potent Anti-Inflammatory Properties of Thalidomide

Among the most adopted mechanisms of action of Thalidomide is its potent anti-inflammatory activity that is achieved by the extensive involvement of both the innate and adaptive immunity. The anti-inflammatory properties of Thalidomide were highly demonstrated in ENL which secured the FDA approval for its usage in treating acute cutaneous manifestations of moderate to severe cases of this disease. Yet, the effectiveness of its anti-inflammatory activity in treating autoimmune diseases (such as rheumatoid arthritis, ulcerative colitis, Crohn's disease) and some dermatological complications was not supported by large-scale randomized clinical trials. Thus, Thalidomide failed to gain a widespread acceptance or an approval from the FDA for its usage in treating these diseases (32).

Numerous *in vitro* and *in vivo* studies in several animal models along with clinical studies on patients have been undertaken to demonstrate the potent anti-inflammatory properties of this drug. As such, Thalidomide was shown to downregulate the phagocytic activity of immune cells, to inhibit the release of antimicrobial mediators from neutrophils, and to enhance the number of natural killer cells (26). Regarding neutrophils, Thalidomide can inhibit their chemotaxis to the site of inflammation, suppress their reactive oxygen species (ROS) generation, and modulate their interaction with the endothelial cells at the site of inflammation (26, 33). As for cytokines and chemokines, Thalidomide has proven to have a key regulatory effect on their production mainly by inhibiting cyclooxygenase enzyme-2 (COX-2) and downregulating soluble levels of mediators such as Prostaglandin E2 (PGE2), TNF-α, IL-1, IL-6 (26). Among the most affected pro-inflammatory cytokines is TNF-α as it was shown to be either degraded at the mRNA level or to be downregulated as a subsequent effect to the inhibited NF-κ β pathway that is highly disrupted by Thalidomide (34). For the adaptive immunity, studies on the impact of Thalidomide on B cells was not well-elaborated, but a demonstrated down regulatory effect on antibody production was supported by the decreased serum IgM concentrations in mice and in leprosy patients (35). As for T-cells, studies on Thalidomide mode of action yielded conflicting results. Thalidomide was thought initially to be associated with increased production of IL-4 and IL-5 and with promoting T-helper cells type 2 (Th2) with the subsequent decrease in IFN-γ production in mitogen- and antigen-stimulated human peripheral blood mononuclear cells (36). Afterwards, an overwhelming amount of data supported its effect on enhancing the differentiation of T-helper cells type 1 (Th1) and the subsequent increase in IFN-γ and IL-2 levels (37). Finally, it was shown that alveolar macrophages of patients with interstitial lung disease reveal a suppressed IL-12 production in response to Thalidomide (26).

## Thalidomide as an Immunomodulatory Drug in Pulmonary Diseases and Lung Injuries

Thalidomide effectiveness was tested in several pulmonary diseases and lung injuries but most of these studies are pre-clinical ones. Among these studies is that concerning the usage of Thalidomide in induced acute lung inflammation by *Klebsiella pneumoniae* in mice. The effective anti-inflammatory activity was presented by the decreased neutrophil influx to the lungs, the suppressed production of malondialdehyde as well as nitric oxide, and the inhibited myeloperoxidase activity (33). Similarly, Thalidomide treatment in mice with Paraquat (PQ) induced pulmonary inflammation and fibrosis revealed a decreased production of inflammatory and fibrogenic cytokines in lung tissues. These included TNF-α, IL-1β, IL-6, TGF-β1 as well as a reduction in myeloperoxidase (MPO), nitric oxide (NO), and hydroxyproline contents which prevented the progression of PQ-induced pulmonary injury (38). Likewise, Thalidomide was able to reduce macrophages, and lymphocytes count in bleomycin (BLM)-induced pulmonary fibrosis mice model and to suppress IL-6, IL-8, TNF-α, and TGF-β levels in their bronchoalveolar lavage fluid (BALF). The substantial attenuation of pulmonary fibrosis and the inhibition of collagen deposition were attributed to the activity of Thalidomide in suppressing inflammation and oxidative stress (39).

Regarding pulmonary viral infections, Thalidomide was able to suppress the induced pulmonary inflammation of H1N1-induced lung injury in mice. The anti-inflammatory activity was achieved through suppressing the expression of cytokines and chemokines released by epithelial and inflammatory cells such as TNF-α, IL-6, RANTES, IFN- α, and IP-10. This inhibition was attributed mainly to the suppressed NF-κ β activity that usually promotes inflammation and viral gene expression (40). Finally only one clinical study featuring 23 patients with Idiopathic pulmonary fibrosis (IPF) treated with Thalidomide reported an improved cough and respiratory quality while the associated side effects were tolerable and included only constipation, dizziness, and malaise (41).

## Thalidomide and COVID-19

**Figure 1:**
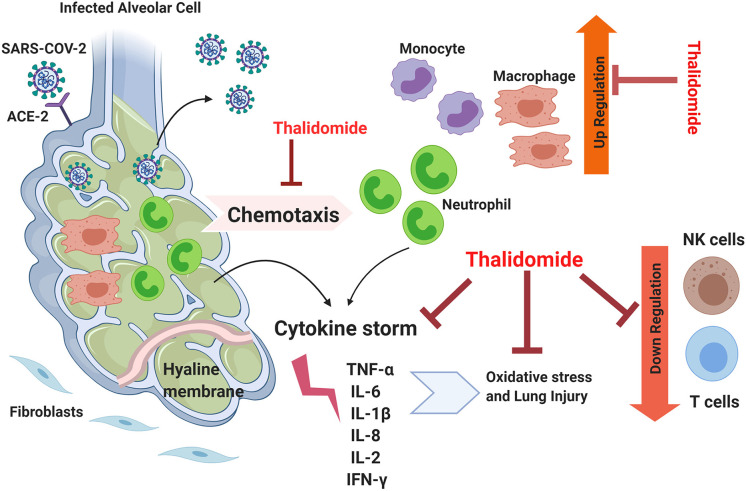
The theoretical efficiency of Thalidomide in attenuating the inflammation associated with COVID-19. Lungs infected by SARS-CoV-2 possess suppressed immune response, elevated inflammation, activated cytokine storm, and excessive oxidation stress leading to lethal lung injury. Thalidomide could potentially inhibit chemotaxis of neutrophils and suppresses them along with that of monocytes. It could possibly downregulate the cytokine storm by acting on several involved factors and can suppress independently the associated oxidative stress. Thalidomide is also known to be an up-regulator for NK and T cells and thus can reverse the downregulatory effect of COVID-19. TNFα, Tumor necrosis factor alpha; IL, interleukin; ACE-2, Angiotensin-converting enzyme 2; IFN-γ, Interferon gamma.

The above cases are characterized by similar disease manifestations, pathogenicity, and progression as the ones encountered in COVID-19 cases. For example, diffuse interstitial lung disease (ILD) is characterized by pulmonary fibrosis that includes inflammation, fibroblast proliferation, and excessive collagen deposition. Since inflammation and oxidative stress are responsible for the high mortality rate associated with this disease, Thalidomide, as an immunomodulatory drug, was proposed as a potential treatment for this lethal condition (39). Similar to COVID-19, Paraquat (PQ) poisoning is known to be associated with respiratory distress due to the alveolar epithelial cell disruption, hemorrhage, and the infiltration of inflammatory cells into the interstitial and alveolar spaces which ends up with fibroblastic proliferation, collagen deposition, and progressive fibrosis. The exaggerated inflammatory process in PQ poisoning is mainly induced by the generation of reactive oxygen species (ROS), induction of intracellular transcription factors such as NF-kB mediators, and the de-regulation of many pro-inflammatory agents including inducible nitric oxide synthase (iNOS), inflammatory cytokines, and cyclooxygenase (38). In both (PQ) and (BLM)-induced pulmonary fibrosis models, the core pro-inflammatory cytokines underlying the pathogenicity of these conditions such as TNF-α, IL-6, IL-1β, and TGF-β are common with that of COVID-19 cases (38, 39). The shared downstream pathway between SARS-CoV2 and H1N1 is that the infected cells can initiate a “cytokine storm,” leading to severe post-infection complications (42). Based on the above, Thalidomide could be hypothetically listed among the potential drugs to be tested in treating respiratory complications associated with COVID-19 based on its potent anti-inflammatory properties and its activity in attenuating exaggerated inflammation and cytokine storms (Figure 1).

## What is the Current Status of Thalidomide in the COVID-19 Crisis?

On February 26th 2020, a case-report preprint manuscript was published online with a single Chinese patient with severe COVID-19 pneumonia being treated with Thalidomide in combination with low-dose glucocorticoids and anti-viral therapy (43). The results presented Thalidomide as a promising therapeutic drug to treat severe cases of COVID-19. The administrated 100 mg of Thalidomide, along with the low dose of methylprednisolone, increased the oxygen index rapidly and suppressed anxiety, nausea, and vomiting in the patient without any reported side effect. This improvement was attributed to the sedative nature of the drug and its antiemetic activities (43). In parallel, the anti-inflammatory and immunoregulatory activity of Thalidomide were associated to the reduced inflammatory cytokines (IL-6, IL-10, and IFN-γ) and the recovered lymphocytes count.

Concomitantly, a couple of phase II clinical trials were registered to evaluate the effectiveness of Thalidomide as an immunomodulatory drug for treating patients with SARS-CoV2 infection. The first clinical trial (NCT04273581) would address the efficacy and safety of this drug in combination with low-dose hormones for treating severe COVID-19 cases. This clinical trial intends to include 40 participants who will be treated with Thalidomide (100 mg/d) along with Methylprednisolone (40 mg, q12h) for 5 days and Abidol (200 mg, 3 times a day) for 7 days to control or relieve lung inflammation. The second trial (NCT04273529) would investigate the efficacy and safety of this drug as an adjuvant treatment for moderate new COVID-19 cases with pneumonia. In this trial, Thalidomide (100 mg) will be used for 14 days to treat lung inflammation in 100 participants with COVID-19 (44, 45).

## Critical Limitations to be Considered When Using Thalidomide in COVID-19 Cases

So far, numerous studies were conducted on the efficiency of Thalidomide in treating hundreds of diseases, yet, the FDA approval remains limited to that of MM and ENL (Table 1) (46). The major limitation toward its adoption as an anti-inflammatory drug for hundreds of the previously tested diseases is not only its questioned efficiency in these conditions but also its undesirable side effects and the associated toxicities. As such, despite being proven highly efficient in some pulmonary inflammatory diseases like severe H1N1-induced pneumonia, and paraquat poisoning lung injury, the studies on Thalidomide in this field were discontinued and stopped at the *in vivo* pre-clinical stage. None of the accumulated results were able to secure the testing of Thalidomide on the above pulmonary diseases at a clinical level. For example, and back to 2014, treating mice infected with H1N1 by Thalidomide resulted in an auspicious outcome, but studies in this area were stopped without any explanation (38, 40, 41). Similarly, the recommendation for using Thalidomide to treat IPF associated cough did not pass the panel vote for treating interstitial lung disease associated cough as per the CHEST guideline methodology (47). Moreover, our group has recently raised concerns about worsening the health condition of lung cancer patients by Thalidomide based on an identified potential molecular target in that context (22, 48, 49). Thus, using this drug for treating respiratory conditions such as those encountered by COVID-19 should be further investigated before proceeding. Moreover, in such cases of severe viral infections, an effective treatment approach should combine both anti-viral and anti-inflammatory activities. This combination can prevent the replication and progression of the virus in the host cells and, at the same time, can suppress the overactive cytokine production and reduces the disease aggravation (50). Thus, since Thalidomide lacks an anti-viral effect, further investigations on its usage should take into consideration combinational approaches to help overcome the virus burden.

Currently, the only available case-report on the efficacy of Thalidomide in treating severe COVID-19 cases is not sufficient to promote the usage of the drug due to several reasons. Aside from being a non-peer reviewed article that describes the outcomes in only one COVID-19 patient, the combination of Thalidomide with corticosteroids might be a drawback since the latter were reported to cause lung injury, and thus, their usage is not clinically supported (20). Second, the two clinical trials that aim at studying the efficacy and safety of this drug in COVID-19 patients were initiated by the same author who published the discussed single case-report. These two trials were registered on February 18th 2020, but none of them has started the recruitment procedure. This delay in initiating such trials at a stage where thousands of severe cases are in need of promising treatment might question Thalidomide potentials in this area. Third, there were no previous studies on the use of Thalidomide in combating the related SARS-CoV2 viruses, namely those that caused SARS and MERS, casting more doubts about its potential. Finally, the known teratogenicity of this drug should be highly taken into consideration when assigning the targeted population who can benefit from this treatment. Thus, further studies on the usage of Thalidomide in COVID-19 cases should take into account the resultant induced birth defects and the severe toxicities that are encountered during its intake, such as sensorimotor peripheral neuropathy, somnolence, acute pulmonary toxicities, and thromboembolic events (22, 51–53). Such restrictions might further hinder investigations in this area since they minimize the potential targeted-population.

## Conclusion

Although the ideal solution for this pandemic remains to be an effective vaccine against COVID-19 or the early destruction of the virus by a new molecule that prevents viral invasion into human cells, these strategies are time-consuming. The rapid progression of this crisis is compelling temporary compensatory actions such as drug repurposing approaches and/or combinational therapies that include anti-inflammatory drugs and anti-viral therapies. Yet, repurposing Thalidomide based on the first glance at its proven efficiency in some pulmonary inflammatory conditions is inadequate, especially if we look in-depth on the reported results and try to question the outcomes of these data at the clinical level. Moreover, when dealing with anti-inflammatory drugs that lack anti-viral activity, like Thalidomide, one should always consider combinational approaches for more promising outcomes.

Although theoretically the anti-inflammatory and the immunomodulatory properties of Thalidomide permit this drug to be a potential candidate for treating the complications of COVID-19, many limitations should be resolved before proceeding into a clinical setting. At this stage, the devastating rapid outcome of COVID-19 is exceptionally granting the utilization of some drugs on the basis of “possible benefits that can outweigh the risk.” However, this urgent need for rapid solution should not certify hasty medical decisions that might lead to an additional man-made crisis. Thus, repurposing some drugs could be beneficial only if an appropriate interpretation of the literature is accompanied by supportive data from pre-clinical studies and well-designed clinical trials.

## Data Availability Statement

The original contributions presented in the study are included in the article/supplementary materials, further inquiries can be directed to the corresponding authors.

## Author Contributions

AKh, AKa, and GN have made significant contributions to writing this manuscript.

## Conflict of Interest

The authors declare that the research was conducted in the absence of any commercial or financial relationships that could be construed as a potential conflict of interest.
